# Motivation for MOOC learning persistence: An expectancy–value theory perspective

**DOI:** 10.3389/fpsyg.2022.958945

**Published:** 2022-08-16

**Authors:** Yechan Lee, Hae-Deok Song

**Affiliations:** ^1^College of Education, Chung-Ang University, Seoul, South Korea; ^2^Department of Education, College of Education, Chung-Ang University, Seoul, South Korea

**Keywords:** MOOC, learning persistence, expectancy–value theory, teaching presence, student engagement

## Abstract

Managing learning continuity is critical for successful MOOC learning. Thus, enabling learners to have learning persistence needs to be integrated into the MOOC learning design. Motivation effort is a critical component enabling students to maintain continuous MOOC learning. The expectancy–value theory explains why learners engage in learning: (1) they have a higher perceived ability for learning success, (2) place value on learning, and (3) avoid psychological costs. However, it is unclear how these factors affect MOOC learning persistence and how learners’ motivation is formed from this perspective. This experimental study explored how learners’ motivational variables affect their learning persistence, focusing on the expectancy–value theory. The results of this study indicated that academic self-efficacy and task value had significant positive effects on learning persistence. The structural relationship of antecedent, process, and outcome variables showed that teaching presence as an antecedent had a significantly positive effect on academic self-efficacy and task value. Among the three factors of the expectancy–value theory, only the task value influenced learning persistence through student engagement as a mediator. Based on the results, suggestions are provided for motivating MOOC environments that support learners’ continuous MOOC learning.

## Introduction

Maintaining learning continuity significantly affects learners’ cognitive, emotional, and behavioral engagement and promotes successful learning. Particularly, in the massive open online courses (MOOCs)—where lectures are started and maintained solely by learners—continuity is a critical indicator in determining successful learning compared to ordinary school education, which is compulsory (Wu and Chen, 2017). In this respect, learning persistence, a concept related to maintaining continuous learning, is imperative to successful MOOC learning.

Given that 90% of MOOC students experience a dropout, many efforts are being conducted to solve this problem (Narayanasamy and Elçi, 2020). Early MOOCs emphasized connections between learners and various platforms, such as connectivist MOOCs (cMOOCs) or openness from classrooms to outside the classrooms in extended MOOCs (xMOOCs). However, owing to the growing emphasis on the individual needs of learners and active feedback, various initiatives in MOOCs have been suggested, such as bended MOOCs (bMOOCs), small MOOCs of a small course or social and seamless MOOCs (sMOOCs), small private online course (SPOC), and the mobile MOOCs (MobiMOOCs) (Bozkurt, 2021; Yousef and Sumner, 2021).

In addition, to enable continuous learning and learning success, studies have examined a variety of variables, such as lesson designs that apply learning analysis (Shukor and Abdullah, 2019), learners’ participation (Yang et al., 2022), quality factors (Gu et al., 2021), or the characteristics of learning environment with technologies (Zhao et al., 2020). These studies have reported that academic skills and abilities, prior experience, social presence/support, course design/complexity/time, and complexity/motivation influence the dropout rate of MOOC learners (Aldowah et al., 2020). Considering that one important variable for successful online learning is self-regulation (Ferguson et al., 2016; Pérez-Sanagustín and Maldonado, 2016), motivation is a key variable as a psychological mechanism enables MOOC learners to select and maintain learning processes voluntarily. Furthermore, MOOC learners are in an environment separated from their instructors and voluntarily chose MOOC learning mainly based on “interest in the course” or “interest in the subject of the lecture” or to obtain a certificate (Milligan and Littlejohn, 2016; Maya-Jariego et al., 2020), and feel burdened by excessive effort, lack of time, or difficult content (Khalil and Ebner, 2014; Eriksson et al., 2017; Bonk et al., 2018). These MOOC environments and the characteristics of learners are related to expectancy–value theory.

In order to understand the learning choices and persistence of MOOC learners, the expectancy–value theory examines how learners comprehensively recognize the “expectancy” that they can successfully complete the learning even in an environment separate from the instructor and the “value” and “cost” of the task. For instance, Bingol et al. (2020) reported that most active MOOC learners performed better when they have strong self-efficacy and interest in the topic as internal motivation factors and sufficient time as an external motivation factor.

However, most of the studies examining MOOCs from the perspective of expectancy–value theory emphasize only one factor—self-efficacy or task value (MacDonald and Ahern, 2015; Jung and Lee, 2018). Furthermore, as a concept distinct from task value, few studies have examined the cost (Zielinski et al., 2019), which is considered to be the main cause of MOOC dropouts. In addition, most of the preceding studies are limited in setting variables, such as expectancy and value as independent variables (Luik et al., 2019; Zielinski et al., 2019), and examining the influences of the variables on outcome variables (Lee et al., 2020; Romero-Rodríguez et al., 2020).

Yo reveal the mechanism of learning motivation on learning persistence and to provide design implications that can be applied to instructors or platform managers, beyond the simple influence relationship between the variables, it is necessary to explore the structural relationship among the variables related to the expectancy–value theory. In this regard, Skinner et al. (2009), who studied the procedure of motivational development, presented the importance of the antecedent variables that affect self-related variables and the process variable that mediates the process where self-related variables lead to learning outcomes. It is possible to understand the whole process of motivation development only through the structural verification of the relationship between variables related to the development and manifestation of motivation. Based on this understanding, intervention can be provided to promote learners’ motivation.

From this perspective, the teacher, parents, fellow learners, and the cultural environment could be considered antecedents affecting learners’ motivation (Eccles et al., 1983). In particular, Hew (2016) suggested interaction with the teacher as one of the five factors to increase the participation and completion rate of MOOC learners, and Khalil and Ebner (2014) stated that MOOC learners consider interaction with teachers as important and that it affects the course satisfaction of the MOOC learners. Thus, it is necessary to examine the teaching presence, which indicates the degree of support provided by the teacher or the interaction with the teacher in the MOOC learning environment, as an antecedent affecting the motivation of the learner from a social cognitive perspective (Aldowah et al., 2020).

Moreover, a process variable should be identified. Skinner et al. (2009), who schematized the motivation development process, emphasized the role of student engagement as a process variable that mediates the learners’ motivation leading to the learning outcomes. Student engagement is related to the active and continuous performance of learners and is a variable that affects learning persistence in a learning environment that requires strong self-directedness, such as in MOOCs (Guajardo Leal and GonzĆ, 2019; Deng et al., 2020). It also reflects learners’ motivation (Alamri, 2022).

Therefore, this study aims to systematically examine the structural relationship between variables that affect learning persistence in the MOOC environment, focusing on academic self-efficacy, task value, and cost, which are sub-factors of expectancy–value theory. To this end, we selected teaching presence as an environmental variable that affects the learners’ expected value, student engagement as a process variable when learners’ motivation leads to learning outcome, and learning persistence as an outcome variable.

### Learning persistence and the expectancy–value theory of the massive open online courses

Learning persistence comprehensively represents learners’ motivation, emotion, cognition, and behavioral factors (Müller, 2008), and it is considered a learning outcome variable of the MOOCs along with the completion rate and intention of completion. Several studies discuss learners’ motivation, educational content, educational support and environment, student engagement, and learning satisfaction as variables affecting learning persistence in the MOOCs (Dai et al., 2020). In particular, recent studies have focused on learning persistence from the perspective of expectancy–value theory, which is known to best predict learners’ decision-making, academic continuity, and learning outcome (Romero-Rodríguez et al., 2020; Chen et al., 2021).

The expectancy–value theory is a motivational theory in that individuals’ expectancy for success and value for tasks is critical to predicting future decisions, participation, continuity, and achievement, and it is primarily composed of two sub-factors: “expectancy” and “task value” (Eccles and Wigfield, 2002). Academic self-efficacy, presented as a more detailed concept than “expectancy for success” by Eccles and Wigfield (2002) in the expectancy–value theory, refers to the learners’ perceived ability to perform a given learning task at the required level (Schunk, 2016). It has been proven to be a factor influencing academic continuity and learning outcome in an online learning environment, such as in the MOOCs, where learners must be able to construct their own learning (Lee et al., 2020).

The task value corresponds to the subjective value that the learner has on the task, and can be classified into four factors depending on its characteristics: attainment value, intrinsic value, utility value, and cost (Eccles et al., 1983). Several studies have found that learners’ perception of task value sustains interest, positively affects learning outcome, and persistence (Valle et al., 2021; Berweger et al., 2022), and are particularly closely related to the reasons for the course selection and continuation of MOOC learners (Milligan and Littlejohn, 2016; Maya-Jariego et al., 2020).

Cost is a perception of the negative aspects of the task (Eccles et al., 1983), and has recently re-emerged as an independent variable rather than a sub-factor of task value (Bergey et al., 2018; Song, 2018). It can be roughly divided into effort, opportunity, and emotional costs (Eccles and Wigfield, 2002) and predicts learners’ avoidance of learning, non-adaptive learning outcome, and dropout in learning situations. It is also related to time management and effort control, which are important for self-regulated learning strategies in an online learning environment (Broadbent and Poon, 2015). This may be a reason for the dropout of MOOC learners (Eriksson et al., 2017).

### Teaching presence as an antecedent of the expectancy–value–cost

Eccles and Wigfield (2002), who re-established the expectancy–value theory from a social cognitive perspective, emphasized the role of socializing agents, such as teachers, parents, and fellow learners, that significantly affect learners as an antecedent focusing on the process of motivational development. In particular, teachers’ roles in MOOCs differ from those in a traditional classroom environment, i.e., they play the role of (1) designers and developers who plan the difficulty, length, and content of online learning; (2) a guide who carries out the learner’s projects; and (3) fellow learners who co-act through learning activities (Lowenthal et al., 2018). From this point of view, social interaction with teachers and social support of teachers are major variables that influence the dropout of MOOC learners (Aldowah et al., 2020). If learners are not well aware of the support of these teachers, they may experience feelings of isolation, frustration, or confusion or may lose interest in class due to the absence of or low interaction with the teachers (Zhang et al., 2018), and it is deemed necessary to consider teaching presence as an antecedent that affects the motivation of the MOOC learners (Yusof et al., 2017).

Teaching presence is defined as “the teacher designs, promotes, and guides learners’ cognitive and social processes so that individual learners can realize teachers’ meaningful efforts in improving learning effects” (Garrison et al., 1999); Teaching presence in the online learning environment positively affected learners’ motivation, participation, satisfaction, outcome, and learning persistence (Turk et al., 2022).

### Student engagement as a mediator of expectancy–value–cost and learning persistence

Skinner et al. (2009) focused on the process of motivation manifestation of the learners and emphasized the importance of student engagement, which plays a mediating role between self-related variables and learning outcomes. In other words, student engagement is a concept in which context and ego are manifested through continuous interest and behavior. It is a noteworthy mediator of motivation development of the MOOC learners such that it explains the mechanism by which the learners’ motivation leads to the learning outcome.

Student engagement refers to “a state in which learners actively concentrate and continuously participate in learning activities to achieve their goals in a learning environment” (Coates, 2006). In several earlier studies, student engagement has been reported as an outcome variable of learning by itself, as well as an antecedent affecting achievement, satisfaction, and learning persistence, and process variable mediating between the learners’ self-related variable and the learning outcome variable (Ferrer et al., 2020; Alamri, 2022). Further, it prevents dropout in the e-learning environment and becomes a motivation to learn repeatedly and continuously. However, if student engagement is not provided in the e-learning environment, the possibility of learning failure due to the dropout and indolence increases (Feklistova et al., 2021). Especially, since it is very difficult to simultaneously promote student engagement of diverse learners in the MOOC learning environment where there is an unprecedented number of students (Hew, 2016), we need to emphasize the student engagement for the learning success of the MOOC learners.

### The current study

**FIGURE 1:**
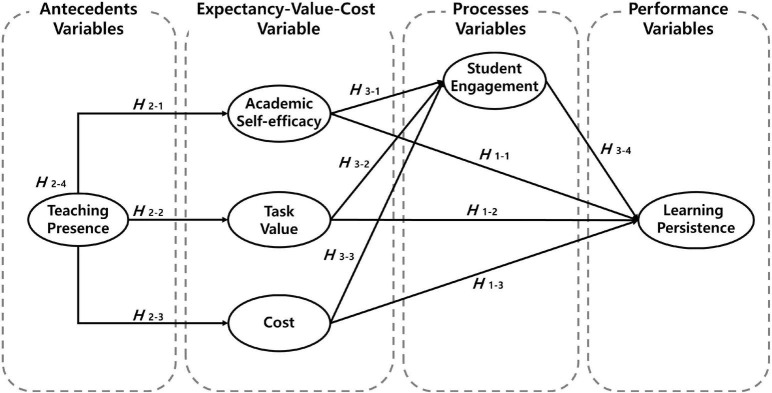
The research model.

In this study, to understand the structural relationship among the sub-variables of the expectancy–value theory that affect learning persistence in the MOOC environment, we *derived research questions by* reviewing the theoretical background and previous studies *and established a research model* as shown in Figure 1.

This study reviewed the theoretical background and previous studies to understand the structural relationship among the sub-variables of the expectancy–value theory that affect learning persistence in the MOOC environment and established a research model as shown in Figure 1. Specifically, we formulated three related hypotheses: (1) academic self-efficacy, task value, and cost will affect learning persistence in the MOOCs. Specifically, academic self-efficacy and task value will have a significant positive effect on learning persistence (H_1–1_, H_1–2_), but the cost will have a significant negative effect on learning persistence (H_1–3_). (2) Teaching presence will affect academic self-efficacy, task value, cost, and learning persistence. Specifically, teaching presence will have a significant positive effect on academic self-efficacy (H_2–1_) and task value (H_2–2_), but have a significant negative effect on the cost (H_2–3_), and teaching presence will affect learning persistence through academic self-efficacy, task value, and cost as mediators (H_2–4_). (3) The student engagement mediates between academic self-efficacy, task value, cost, and learning persistence. Specifically, academic self-efficacy and task value will have a significant positive effect on student engagement (H_3–1_, H_3–2_), but the cost will have a significant negative effect on student engagement (H_3–3_), and the student engagement will multi-mediate between teaching persistence, academic self-efficacy, task value, cost, and learning persistence (H_3–4_).

## Materials and methods

### Participants and research context

An online survey was conducted on students from two courses loaded on a Korean-MOOC (K-MOOC) in 2020. Among the 298 questionnaires retrieved, 277 responses were finally used for the analysis, excluding duplicate participation, non-response, and identical responses. The K-MOOC course sampled by the research context consisted of 14 weeks of lectures covering the field of education and was provided free of charge to the general public on the K-MOOC platform. The courses consist of 3–5 lecture videos of 10–20 min per week and learning activities, such as quizzes, discussions, or reports. To issue a certificate of completion, learners must achieve a certain level of scores through quizzes and participate in discussions or writing reports; however, this was not linked to their college credit.

The courses are operated as a regular 14-week course (cardinal) rather than a full-time system (regular), and the instructor and tutor interact with the learner, while the lecture is in operation. Specifically, when the weekly lecture starts, the instructor emails the learner a greeting and a guide about the class content for the week. In addition, the tutor participates in the discussion to promote it so that learners are encouraged to provide varied opinions. When learners ask a question, the instructor and tutor answer within 24 h, and feedback is provided for the submitted assignment.

### Instruments

A questionnaire was constructed as a measurement tool for teaching presence, academic self-efficacy, task value, cost, student engagement, and learning persistence, verified through previous studies. The survey was modified and adapted to the MOOC environment based on the advice of experts in educational technology, and the reliability and validity of the tool were verified based on the results of confirmatory factor analysis and exploratory factor analysis.

To measure the teaching presence, we modified and used a tool developed by Swan et al. (2008). This instrument consists of 13 items (e.g., “The instructor helped keep course participants engaged and participate in productive dialog”) on a five-point Likert-type scale. For academic self-efficacy, we used the motivated strategies for learning questionnaire (MSLQ) (Pintrich and De Groot, 1990; Bong, 2008; Jung and Lee, 2018). This instrument consists of five items (e.g., “I expect to do very well in this class.”) on a five-point Likert-type scale. For the task value, we modified and used tools developed by Eccles et al. (1984), and validated and translated by Joo et al. (2013) to suit the MOOC environment. This instrument consists of six items on a five-point Likert-type scale, and the sample items are “In general, what I learn in class is useful in real life” for utility value; “It is important to learn successfully in this class” for attainment value; “I enjoy learning in this class” for intrinsic value. For the cost, we modified and used tools developed and validated by Jiang (2015) to suit the MOOC environment. This instrument consists of nine items on a seven-point Likert-type scale, and the sample items are “It requires too much effort for me to get a good grade in MOOC class” for effort cost; “I have to manage considerable free time of MOOC class” for opportunity cost; and “Taking MOOC class makes me feel stressed” for emotional cost. For the student engagement, we modified and used the tools developed by Deng et al. (2020), which measure learning engagement in the MOOCs, according to the results of exploratory factor analysis and confirmatory factor analysis and the characteristics of the *research context.* This instrument consists of eight items on a six-point Likert-type scale, and the sample items are “I took notes while studying the MOOC” for behavioral engagement; “I was inspired to expand my knowledge in the MOOC” for emotional engagement; “When I had trouble understanding a concept or an example, I went over it again until I understood it” for cognitive engagement. For learning persistence, we modified and used the tool developed by Shin (2003), and constructed the tool by removing items that did not meet the significance criteria based on the exploratory factor analysis. This instrument consists of three items (e.g., “I will finish my studies at this MOOC class no matter how difficult it may be.”) on a five-point Likert-type scale.

### Data analysis

This study adopts a quantitative cross-sectional research design to examine the relationships between the variables that affect the learning persistence of MOOC learners (Creswell, 2012). The main statistical technique for analyzing the collected data is the analysis of the structural equation model that can analyze several independent variables simultaneously and can estimate the moderating effect and the mediating effect. The analysis was conducted using SPSS 26.0 and AMOS 26.0 statistical software.

First, frequency analysis was performed to confirm the demographic characteristics of the survey respondents. To test the normality of the data, we conducted a descriptive statistical analysis and examined the mean, standard deviation (≥ 0.150, Meir and Gati, 1981), skewness, and kurtosis (| skewness| ≤ 2.000, | kurtosis| ≤ 2.000, Bandalos and Finney, 2010) of the variables, and conducted Pearson’s correlation analysis (*p* < 0.05) to confirm the correlation between the variables.

Then, we conducted verification of the construct validity of the measurement tool through exploratory factor analysis (communalities < 0.40, factor loading < 0.40, Mat Roni, 2014) and item parceling to prevent excessive weight from being applied to the measurement model. Further, Harman’s single factor test was conducted to confirm the effect of the common method bias (total variance < 50%, Mat Roni, 2014).

Next, we examined the fit indices, such as χ^2^, TLI, CFI (≥ 0.90), SRMR (≤ 0.08), RMSEA (≤ 0.08), reliability (Cronbach α ≥ 0.60) (Hu and Bentler, 1999), and convergent validity (standardized regression weights ≥ 0.50, average variance extracted ≥ 0.50, overall reliability ≥ 0.50, Hair et al., 2010) and discriminant validity [AVE > *r*^2^, *r* ± (2*SE)].

Finally, to check the significant relationship between the variables in the research model, we examined the direct effect, statistical significance (*p* < 0.05) for the direct, indirect, and total effect by setting bias-corrected 95% confidence interval for the estimates derived through a total of 5,000 bootstrapping, and the indirect effect of individual pathways in the dual mediation through the phantom model (Macho and Ledermann, 2011).

## Results

### Descriptive statistics and correlations between the variables

We tested the multivariate normality of the data collected in this study and conducted a correlation analysis to confirm the association between the study variables. The analysis results are shown in Table 1.

**TABLE 1:** Measurement items.

Measurement variable	Mean	SD	1	2	3	4	5	6
1. Academic Self-efficacy	4.056	0.570	1					
2. Task Value	3.950	0.607	0.622**	1				
3. Cost	3.277	1.078	−0.332**	−0.212**	1			
4. Teaching Presence	3.995	0.631	0.472**	0.469**	−0.164**	1		
5. Student Engagement	4.497	0.767	0.419**	0.540**	−0.127*	0.417**	1	
6. Learning Persistence	4.013	0.553	0.592**	0.597**	−0.286**	0.457**	0.514**	1

*p < 0.05, **p < 0.01.

The means of the variables range from 3.277 to 4.497, and the standard deviations range from 0.589 to 1.49, satisfying the criteria of relevance. The absolute values of skewness and kurtosis were analyzed to test the normality of variables: the absolute value of skewness ranges from 0.090 to 0.917 and the absolute value of kurtosis from 0.008 to 0.875, which are all deemed to satisfy the normality requirement (Kline, 2011). The result of the correlation analysis showed that significant correlations existed between all variables. The correlation coefficients between the variables are all in the range of 0.127–0.622 in absolute values, indicating that no issue arises from multicollinearity (Grewal et al., 2004).

### Assessing the measurement model

Regarding the measurement model designed in this study, the goodness of fit index obtained due to the confirmatory factor analysis was calculated as TLI = 0.924, CFI = 0.942, SRMR = 0.064, and RMSEA = 0.065, indicating that the measurement model is plausible. The measurement model was evaluated based on reliability, convergent validity, and discriminant validity. Reliability can be confirmed through the Cronbach α coefficient. The Cronbach α value of each variable is found to be 0.758–0.937, confirming that all variables met the reliability criterion (≥ 0.60). Convergent validity can be verified through factor loading, average variance extracted (AVE), and overall reliability. The analysis showed that the standardized factor loading for each variable is 0.534–0.953 (≥ 0.50, Hair et al., 2010), the AVE is 0.513–0.667 (≥ 0.50, Fornel and Larcker, 1981), and the overall reliability is 0.737–0.856 (≥ 0.50, Hair et al., 2010), indicating that the convergent validity of the measurement variables is adequate. In the case of discriminant validity, if the AVE is greater than the squared value of the correlation coefficient between each latent variable, then the model is considered to satisfy a criterion (Fornel and Larcker, 1981), and if this criterion is not satisfied, it is judged that there is discriminant validity if the result of r ± (2*SE) calculation does not include 1 through additional analysis (Anderson and Gerbing, 1988). The analysis showed that the square value of the correlation coefficient between most variables was smaller than the corresponding AVE, and the result of calculating r ± (2*SE) of the relationship between variables that did not meet this criterion (between academic self-efficacy and learning persistence, between the task value and learning persistence) did not include 1, indicating that the discriminant validity of the measurement variables is adequate.

### Assessment of structural model

Regarding the structural model designed in this study, most goodness of fit criteria were satisfied (TLI = 0.896, CFI = 0.917, SRMR = 0.076, and RMSEA = 0.070), so we decided that this structural model is plausible. However, according to the result of parameter estimation on the influential relationship between the latent variables, it was found that academic self-efficacy (*B* = 0.044, β = 0.035, *p* > 0.5) and cost (*B* = 0.037, β = 0.083, *p* > *0.5*) did not have statistically significant effects on the student engagement. So, we modified the model to construct a simpler one by deleting two direct paths that are not statistically significant from the initial structural model (H_3–1_, H_3–3_), and by proving that the initial model and modified model do not show a statistically significant difference (Δχ^2^ = 1.830, Δ*df* = 2, *p* = 0.401).

**FIGURE 2:**
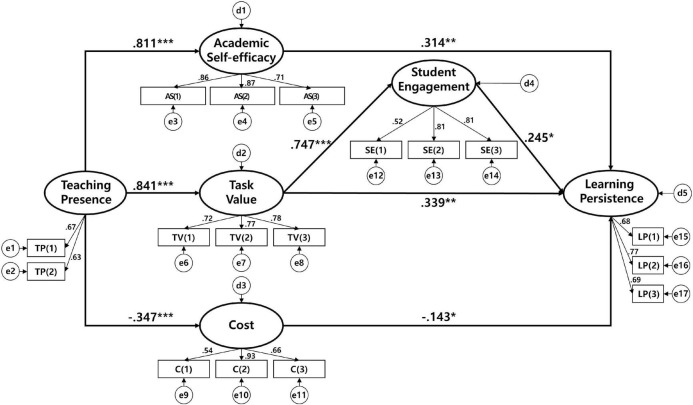
The research findings.

Therefore, as shown in Figure 2, the simpler modified model was adopted as the final model, and the direct effects between the variables of the final model are shown in Table 2.

**TABLE 2:** Total, direct, and indirect effects among variables.

			Total effect (β)	Direct effect (β)	Indirect effect (β)
Teaching Presence	→	Academic Self-efficacy	0.811***	0.811***	–
	→	Task Value	0.841***	0.841***	–
	→	Cost	−0.347**	−0.347**	–
	→	Student Engagement	0.628***	–	0.628***
	→	Learning Persistence	0.743***	–	0.743***
Academic Self-efficacy	→	Student Engagement	–	–	–
	→	Learning Persistence	0.314*	0.314**	–
Task Value	→	Student Engagement	0.747***	0.747***	–
	→	Learning Persistence	0.522***	0.339*	0.183*
Cost	→	Student Engagement	–	–	–
	→	Learning Persistence	−0.143*	−0.143*	–
Student Engagement	→	Learning Persistence	0.245*	0.245*	–

*p < 0.05, **p < 0.01, ***p < 0.001.

First, academic self-efficacy, task value, and cost had statistically significant effects on learning persistence. Second, teaching presence had statistically significant effects on academic self-efficacy, task value, and cost. Third, the direct effect of task value on student engagement and student engagement was statistically significant. Therefore, the hypotheses H_1–1_, H_1–2_, H_1–3_ and H_2–1_, H_2–2_, H_2–3_ were supported.

### Mediation analysis

According to the results of mediating effect analysis, as shown in Table 2 the indirect effect of the task value on learning persistence (β = 0.183, *p* < 0.05) was found to be significant, and that of teaching presence on student engagement (β = 0.628, *p* < 0.000) and learning persistence (β = 0.743, *p* < 0.000) were also found to be significant. The goodness of fit of the phantom model for the individual path analysis between teaching presence and learning persistence was consistent with that of the final structural model (χ^2^ = 285.519, *df* = 111, TLI = 0.898, CFI = 0.917, SRMR = 0.075, RMSEA = 0.070). Additionally, as shown in Table 3 the individual mediating effects of the four pathways were statistically significant, and the sum of the individual mediating effects was consistent with the indirect effect of the teaching presence on learning persistence. Through this, the mediating effect of academic self-efficacy, task value, and cost between teaching presence and learning persistence and the multi-mediating effect of student engagement was proved, supporting the hypothesis H_2–4_ while partially supporting H_3–4_.

**TABLE 3:** Individual mediating effect analysis results between teaching presence and learning persistence.

Path	β
Teaching Presence → Academic Self-efficacy → Learning Persistence	0.255**
Teaching Presence → Task Value → Learning Persistence	0.285**
Teaching Presence → Task Value → Student Engagement → Learning Persistence	0.154*
Teaching Presence → Cost → Learning Persistence	0.050*
Teaching Presence’s Indirect effect on Learning Persistence:(0.255) + (0.285) + (0.154) + (0.050) = 0.744***	

*p < 0.05, **p < 0.01, ***p < 0.001.

## Discussion and conclusion

### Discussion

This study explored the structural relationships between variables that affect learners’ persistence for MOOC learning from expectancy–value theory. To this end, we selected teaching presence as an environmental variable that affects the learners’ expected value, student engagement as a process variable when learners’ motivation leads to learning outcome, and learning persistence as an outcome variable. Findings show the following points:

First, the MOOC learning environment, learners’ academic self-efficacy, task value, and cost significantly affected learning persistence. The positive effect of academic self-efficacy on learning persistence means that the academic self-efficacy of learners can increase the intention to continue learning by enabling learners to gain self-confidence in the MOOC learning environment. Therefore, instructors should use instructional design strategies to promote learners’ academic self-efficacy, and self-regulated learning strategies can be one way of doing this (Shea and Bidjerano, 2010). Further, their self-efficacy may be improved by utilizing learning analysis dashboard-based self-regulation facilitation tools, such as the NoteMyProgress (NMP) tool (Pérez-Álvarez et al., 2020).

Next, the positive effect of task value on learning persistence is related to learners’ motivation to take the MOOCs (Wu et al., 2020; Moore and Wang, 2021). In other words, the importance, usefulness, and interest that learners feel about the task can explain several motivations for taking and continuing the MOOCs, and task value has a great influence on learning persistence. So, the instructor should make the learners feel interesting and important about the task by linking real-life phenomena to the lecture content or capturing the latest trends and issues on related topics. And also, the instructor can award differentiated certificates to them by subdividing and specifying course completion standards and evaluation standards.

Interestingly, unlike previous studies, this study focused on the cost perceived by the MOOCs and confirmed that the cost had a negative effect on the learning persistence, which was related to the dropout of the MOOC learners. This shows that the demand for excessive effort and time was the reasons for the dropout (Khalil and Ebner, 2014; Eriksson et al., 2017), which means that the demand for efforts and time and opportunity cost that learners experience during the MOOC learning process lowers learning persistence. This suggests that the cost of the task recognized by the learners needs to be lowered to increase the learning persistence. As a strategy to lower the cost for learners, the instructor can reduce the burden felt at the beginning of a lecture by adjusting the difficulty level of the task to be gradually increased or to use a scaffolding strategy by identifying the problems the learner is having difficulty with (Borrella et al., 2021).

Second, teaching presence as an antecedent variable had a significant effect on learners’ academic self-efficacy, task value, and cost; through these, it also had a significant effect on learning persistence. It means that, when learners feel that they can receive appropriate help from the instructors, learners earn confidence to do well in the MOOC learning process, and feel less pressure from the amount of effort they need to put into the MOOC learning process. The significant effect of teaching presence can be explained by the high perception of teachers’ roles and preparation levels in this study. MOOC teachers in this study played the role of designers, developers, and guides. They also played fellow learners during discussions between learners. Both the instructor and tutors in this study have been teaching the course for the past 3 years. To promote a teaching presence, teachers’ active interaction and professional class preparation must be encouraged. In addition, their electronic teaching skills should be improved. Teachers often face difficulties in MOOC development due to a lack of teaching experience in an online environment or technical difficulties, such as filming and editing (Blackmon, 2018; Lowenthal et al., 2018). Further, through the interaction with the instructors, students can be less affected by negative emotions, such as isolation and alienation. Therefore, a strategy to promote the teaching presence of the MOOC learners is required. For example, an instructor can create additional lecture videos based on students’ questions or interact with them in an asynchronous discussion. This will allow a variety of learners to participate without burden, or provide real-time feedback and emotional connection through live lectures or Google Hangouts (Goshtasbpour et al., 2021; Zhu, 2022).

Third, student engagement as a process variable mediated only the relationship between task value and learning persistence among academic self-efficacy, task value, and cost in the MOOC environment. It means that, the more the learners feel the MOOC learning process is important and useful, the more time and money they invest in the task, resulting in behavioral, emotional, and cognitive engagement (Putwain et al., 2019; Romero-Rodríguez et al., 2020). Especially, the intrinsic value is a variable highly related to the learners’ emotional engagement; thus, the more the learners become interested and attracted to the tasks in the MOOCs, the more they become emotionally engaged, forming a sense of belonging to the MOOC learning and bonding with the instructors and fellow learners, and becoming more cognitively and behaviorally engaged. And, in a learning environment that requires learners’ self-directedness, such as in the MOOCs, the student engagement, which indicates active participation of learners, predicts academic continuity, academic achievement, and academic satisfaction more strongly (Yusof et al., 2017).

Therefore, the establishment of interactive, inferential, integrative, and involving instructional design strategies that promote student engagement is required (Hsu et al., 2021). Moreover, the instructor can use a learning analytic tool, such as edX-LIS, which is providing feedback on learners’ performance based on log data (Cobos and Ruiz-Garcia, 2021).

Unlike the previous studies (Jung and Lee, 2018), academic self-efficacy and cost were found to have no statistically significant effects on student engagement. This result was derived even though academic self-efficacy has a high correlation with student engagement because the task value has a relatively large influence on student engagement, and this is similar to the study of Song (2018), which verified the relative predictive power of cost and self-efficacy on career choice and learning participation in the mathematics education. The fact that cost does not have a statistical effect on student engagement can be explained as a contextual feature of the MOOC learning environment. Unlike ordinary school education, which has a certain degree of compulsion in the learners’ class selection and learning continuity, the MOOC learning environment has no special cost, effort, or disadvantage in choosing or giving up a course. Thus, in a learning environment, such as in the MOOCs, if the cost perceived by learners is high, they tend to immediately give up on the task, meaning that the cost has a direct negative effect on learning persistence without going through student engagement. As such, because the cost of the MOOC learners leads to the choice of immediate task abandonment, instructional design and systematic support are needed to be guaranteed to ease the burden on learners in the MOOC learning process.

### Conclusion

Managing learning continuity is critical for successful MOOC learning. Thus, motivation efforts that enable learners to have learning persistence need to be integrated into the MOOC learning design. In this study, to explore learners’ motivation for MOOC learning persistence, the environmental, motivation, and process variables of MOOC learners were selected from the expectancy–value theory. Previous studies on MOOC learning from the viewpoint of motivation mainly focused on learners’ psychological characteristics, such as self-determination theory (Semenova, 2020) or self-regulated learning strategies (Albelbisi et al., 2021). However, given that considering the MOOC context or the reason for the attendance and dropout of the learner, their expectations and values need to be considered. In this respect, this study applied the perspective of expectancy–value theory to the MOOC environment and explored how learners’ expectations and values are formed and expressed in the MOOC learning environment and how it affects learning persistence. Also, with regard to costs not considered in previous studies, it was confirmed that they had a direct and immediate negative effect on learning persistence, proving that there is a need to pay attention to the costs perceived by learners to maintain their MOOC learning.

In addition, previous studies that have mainly analyzed the relationship between the expectancy–value variable and the learning outcome variable were only fragmentary. This approach has limitations in understanding and facilitating learners’ motivational mechanisms, so it is necessary to look at the motivational development process from an integrative point of view (Skinner et al., 2009). This study differs from previous studies in that it established the procedure of motivational development by examining the structural relationship of the environmental, self-related, process, and performance variables and comprehensively analyzed the relationship between them to view the process of motivation manifestation of the learners in the MOOC environment.

Through this study, the importance of teaching presence was highlighted as an antecedent variable affecting the expectancy, value, and cost for MOOC learners. This provides implications for the theoretical foundation and development direction of AI tutor or conversational agent systems related to providing customized feedback on the learners’ responses (González-Castro et al., 2021), or the course recommendation according to the learning status and preference of the learner (Kim and Kim, 2020) in the MOOC environment.

In addition, student engagement as a process variable mediated only the relationship between task value and learning persistence in this study. So, to maintain continuous MOOC learning in MOOC learning design, it is necessary to confirm if learners’ perceptions of task value sustain interest and if their engagement is provided in the MOOC learning environment.

This study has great significance in that we derived practical implications for the development of prescriptive teaching and learning strategies for the formation and expression of the expectancy–value from a holistic view on the process of motivational development. This suggests that to increase the MOOC learners’ learning persistence, it is important to establish an instructional design, create a learning environment, and build a system that comprehensively considers the learners’ teaching presence, academic self-efficacy, task value, cost, and student engagement.

### Limitations and future directions

Our study has limitations. First, in the case of MOOC courses operated in Korea (K-MOOC), there is a tendency for MOOC certificates not to be recognized as work experience, as most courses are provided for free. Conversely, in other countries, MOOC completion is recognized in careers when getting a job or promotion, despite MOOC courses charging a fee in some cases. Depending on these countries and cultures, the value and cost perceived by learners of MOOC courses may have different effects on student engagement and learning persistence.

With respect to the scope for research variables, this study examined the effect of motivation variables on learning persistence from an integrated perspective. However, it is hard to understand the learners’ motivation and engagement in detail in this way. Therefore, in a smaller scope, it is necessary to select each of the sub-factors constituting an individual variable as one potential variable (e.g., task value: achievement value, effort value, and useful value), examine the influential relationship between the variables, cluster according to the level of differences between the variables, and finally explore the characteristics of the group and the differences from other groups. Through this, it will be possible to provide basic data for the provision of customized learning diagnosis and instructional treatment for the learners by understanding learners’ motivation and the level of engagement in-depth and classifying the learning types based on the level of expectancy–value.

Finally, with respect to measurement and research methods, the variables in this study were limited as they were measured through a self-reporting questionnaire. Considering that all the learning processes of learners are stored as big data on the platform due to the nature of the MOOC environment, learning analytics research such as log data analysis, text mining, and social networking analysis can be conducted. Through this, it can be possible to analyze the learning participation patterns of the MOOC learners and provide basic data for the development of a learning management system based on user interface and AI-applied learning tutors.

## Data availability statement

The raw data supporting the conclusions of this article will be made available by the authors, without undue reservation.

## Ethics statement

Ethical review and approval was not required for the study on human participants in accordance with the local legislation and institutional requirements. Written informed consent for participation was not required for this study in accordance with the national legislation and the institutional requirements.

## Author contributions

All authors listed have made a substantial, direct, and intellectual contribution to the work and approved it for publication.

## References

[B1] Alamri M. M. (2022). Investigating students’ adoption of MOOCs during COVID-19 pandemic: students’ academic self-efficacy, learning engagement, and learning persistence. *Sustainability* 14:714. 10.3390/su14020714

[B2] Albelbisi N. A. Al-Adwan A. S. Habibi A. (2021). Self-regulated learning and satisfaction: a key determinants of MOOC success. *Educ. Inf. Technol.* 26 3459–3481. 10.1007/s10639-020-10404-z

[B3] Aldowah H. Al-Samarraie H. Alzahrani A. I. Alalwan N. (2020). Factors affecting student dropout in MOOCs: a cause and effect decision-making model. *J. Comput. High. Educ.* 32 429–454. 10.1007/s12528-019-09241-y

[B4] Anderson J. C. Gerbing D. W. (1988). Structural equation modeling in practice: a review and recommended two-step approach. *Psychol. Bull.* 103 411–423. 10.1037/0033-2909.103.3.411

[B5] Bandalos D. L. Finney S. J. (2010). “Factor analysis: exploratory and confirmatory,” in *The Reviewer’s Guide to Quantitative Methods in the Social Sciences*, eds Hancock G. R. Mueller R. O. (New York, NY: Routledge), 93–114.

[B6] Bergey B. W. Parrila R. K. Deacon S. H. (2018). Understanding the academic motivations of students with a history of reading difficulty: an expectancy-value-cost approach. *Learn. Individ. Differ.* 67 41–52. 10.1016/j.lindif.2018.06.008

[B7] Berweger B. Born S. Dietrich J. (2022). Expectancy-value appraisals and achievement emotions in an online learning environment: within-and between-person relationships. *Learn. Instr.* 77:101546. 10.1016/j.learninstruc.2021.101546

[B8] Bingol I. Kursun E. Kayaduman H. (2020). Factors for success and course completion in massive open online courses through the lens of participant types. *Open Prax.* 12 223–239. 10.5944/openpraxis.12.2.1067

[B9] Blackmon S. (2018). MOOC makers: professors’ experiences with developing and delivering MOOCs. *Int. Rev. Res. Open Distrib. Learn.* 19 76–91. 10.19173/irrodl.v19i4.3718

[B10] Bong M. (2008). Effects of parent-child relationships and classroom goal structures on motivation, help-seeking avoidance, and cheating. *J. Exp. Educ.* 76 191–217. 10.3200/JEXE.76.2.191-217

[B11] Bonk C. J. Zhu M. Kim M. Xu S. Sabir N. Sari A. R. (2018). Pushing toward a more personalized MOOC: exploring instructor selected activities, resources, and technologies for MOOC design and implementation. *Int. Rev. Res. Open Distrib. Learn.* 19 92–115. 10.19173/irrodl.v19i4.3439

[B12] Borrella I. Caballero-Caballero S. Ponce-Cueto E. (2021). Taking action to reduce dropout in MOOCs: tested interventions. *Comput. Educ.* 179:104412. 10.1016/j.compedu.2021.104412

[B13] Bozkurt A. (2021). Surfing on three waves of MOOCs: an examination and snapshot of research in massive open online courses. *Open Prax.* 13 296–311. 10.5944/openpraxis.13.3.132

[B14] Broadbent J. Poon W. L. (2015). Self-regulated learning strategies & academic achievement in online higher education learning environments: a systematic review. *Internet High. Educ.* 27 1–13. 10.1016/j.iheduc.2015.04.007

[B15] Chen C. Sonnert G. Sadler P. M. Malan D. J. (2021). Foreseeing the endgame: who are the students who take the final exam at the beginning of a MOOC? *Behav. Inf. Technol.* 40 565–577. 10.1080/0144929X.2019.1711452

[B16] Coates H. (2006). *Student Engagement in Campus-Based and Online Education: University Connections.* New York, NY: Routledge. 10.4324/9780203969465

[B17] Cobos R. Ruiz-Garcia J. C. (2021). Improving learner engagement in MOOCs using a learning intervention system: a research study in engineering education. *Comput. Appl. Eng. Educ.* 29 733–749. 10.1002/cae.22316

[B18] Creswell J. W. (2012). *Educational Research: Planning, Conducting, and Evaluating Quantitative and Qualitative Research*, 4th Edn. Boston, MA: Pearson.

[B19] Dai H. Teo T. Rappa N. A. (2020). Understanding continuance intention among MOOC participants: the role of habit and MOOC performance. *Comput. Hum. Behav.* 112:106455. 10.1016/j.chb.2020.106455

[B20] Deng R. Benckendorff P. Gannaway D. (2020). Learner engagement in MOOCs: scale development and validation. *Br. J. Educ. Technol.* 51 245–262. 10.1111/bjet.12810

[B21] Eccles J. S. Adler T. F. Meece J. L. (1984). Sex differences in achievement: a test of alternate theories. *J. Pers. Soc. Psychol.* 46 26–43. 10.1037/0022-3514.46.1.26

[B22] Eccles J. S. Adler T. F. Futterman R. Goff S. B. Kaczala C. M. Meece J. L. (1983). “Expectancies, values, and academic behaviors,” in *Achievement and Achievement Motives: Psychological and Sociological Approaches*, ed. Spence J. T. (San Francisco, CA: W. H. Freeman), 75–146.

[B23] Eccles J. S. Wigfield A. (2002). Motivational beliefs, values, and goals. *Annu. Rev. Psychol.* 53 109–132. 10.1146/annurev.psych.53.100901.135153 11752481

[B24] Eriksson T. Adawi T. Stöhr C. (2017). “Time is the bottleneck”: a qualitative study exploring why learners drop out of MOOCs. *J. Comput. High. Educ.* 29 133–146. 10.1007/s12528-016-9127-8

[B25] Feklistova L. Lepp M. Luik P. (2021). Learners’ performance in a MOOC on programming. *Educ. Sci.* 11:521. 10.3390/educsci11090521

[B26] Ferguson R. Scanlon E. Harris L. (2016). Developing a strategic approach to MOOCs. *J. Interact. Media Educ.* 2016:21. 10.5334/jime.439

[B27] Ferrer J. Ringer A. Saville K. Parris M. A. Kashi K. (2020). Students’ motivation and engagement in higher education: the importance of attitude to online learning. *High. Educ.* 83 317–338. 10.1007/s10734-020-00657-5

[B28] Fornel C. Larcker D. F. (1981). Evaluating structural equation models with unobservable variables and measurement error. *J. Mark. Res.* 18 39–50. 10.1177/002224378101800104

[B29] Garrison D. R. Anderson T. Archer W. (1999). Critical inquiry in a text-based environment: computer conferencing in higher education. *Internet High. Educ.* 2 87–105. 10.1016/S1096-7516(00)00016-6

[B30] González-Castro N. Muñoz-Merino P. J. Alario-Hoyos C. Kloos C. D. (2021). Adaptive learning module for a conversational agent to support MOOC learners. *Australas. J. Educ. Technol.* 37 24–44. 10.14742/ajet.6646

[B31] Goshtasbpour F. Swinnerton B. J. Pickering J. D. (2021). Twelve tips for engaging learners in online discussions. *Med. Teach.* 44 244–248. 10.1080/0142159X.2021.1898571 33730976

[B32] Grewal R. Cote J. A. Baumgartner H. (2004). Multicollinearity and measurement error in structural equation models: implications for theory testing. *Mark. Sci.* 23 519–529. 10.1287/mksc.1040.0070 19642375

[B33] Gu W. Xu Y. Sun Z. J. (2021). Does MOOC quality affect users’ continuance intention? Based on an integrated model. *Sustainability* 13:12536. 10.3390/su132212536

[B34] Guajardo Leal B. E. GonzĆ V. (2019). Student engagement as a predictor of xMOOC completion: an analysis from five courses on energy sustainability. *Online Learn. J.* 23 105–123. 10.24059/olj.v23i2.1523 33692645

[B35] Hair J. F. Black W. C. Babin B. J. Anderson R. E. (2010). *Multivariate Data Analysis*, 7th Edn. Upper Saddle River, NJ: Prentice-Hall.

[B36] Hew K. F. (2016). Promoting engagement in online courses: what strategies can we learn from three highly rated MOOCs. *Br. J. Educ. Technol.* 47 320–341. 10.1111/bjet.12235

[B37] Hsu W. C. Garimella V. B. Lee L. (2021). “Examining the factors that affect online learning engagement: a micro-qualitative approach,” in *Learning How to Learn Using Multimedia* (Singapore: Springer), 11–22. 10.1007/978-981-16-1784-3_2

[B38] Hu L. T. Bentler P. M. (1999). Cutoff criteria for fit indexes in covariance structure analysis: conventional criteria versus new alternatives. *Struct. Equ. Model.* 6 1–55. 10.1080/10705519909540118

[B39] Jiang Y. (2015). *The Role of Perceived Cost in Students’ Academic Motivation and Achievement.* Doctoral dissertation. Seoul: Korea University.

[B40] Joo Y. J. Lim K. Y. Kim J. (2013). Locus of control, self-efficacy, and task value as predictors of learning outcome in an online university context. *Comput. Educ.* 62 149–158. 10.1016/j.compedu.2012.10.027

[B41] Jung Y. Lee J. (2018). Learning engagement and persistence in massive open online courses. *Comput. Educ.* 122 9–22. 10.1016/j.compedu.2018.02.013

[B42] Khalil H. Ebner M. (2014). “MOOCs completion rates and possible methods to improve retention–a literature review,” in *Proceedings of the World Conference on Educational Multimedia, Hypermedia and Telecommunications 2014* (Chesapeake, VA: AACE), 1236–1244.

[B43] Kim W. H. Kim J. H. (2020). Individualized AI tutor based on developmental learning networks. IEEE Access, 8, 27927–27937. 10.1109/ACCESS.2020.2972167

[B44] Kline R. B. (2011). *Principles and Practice of Structural Equation Modeling*, 3rd Edn. New York, NY: The Guilford Press.

[B45] Lee D. Watson S. L. Watson W. R. (2020). The relationships between self-efficacy, task value, and self-regulated learning strategies in massive open online courses. *Int. Rev. Res. Open Distance Learn.* 21 1–22. 10.19173/irrodl.v20i5.4389

[B46] Lowenthal P. Snelson C. Perkins R. (2018). Teaching massive, open, online, courses (MOOCs): tales from the front line. *Int. Rev. Res. Open Distrib. Learn.* 19 1–18. 10.19173/irrodl.v19i3.3505

[B47] Luik P. Suviste R. Lepp M. Palts T. Tõnisson E. Säde M. (2019). What motivates enrolment in programming MOOCs? *Br. J. Educ. Technol.* 50 153–165. 10.1111/bjet.12600

[B48] MacDonald P. Ahern T. C. (2015). Exploring the instructional value and worth of a MOOC. *J. Educ. Comput. Res.* 52 496–513. 10.1177/0735633115571927 35756360

[B49] Macho S. Ledermann T. (2011). Estimating, testing, and comparing specific effects in structural equation models: the phantom model approach. *Psychol. Methods* 16 34–43. 10.1037/a0021763 21299303

[B50] Mat Roni S. (2014). *Introduction to SPSS.* Joondalup WA: Edith Cowan University, SOAR Centre.

[B51] Maya-Jariego I. Holgado D. González-Tinoco E. Castaño-Muñoz J. Punie Y. (2020). Typology of motivation and learning intentions of users in MOOCs: the MOOCKNOWLEDGE study. *Educ. Technol. Res. Dev.* 68 203–224. 10.1007/s11423-019-09682-3

[B52] Meir E. I. Gati I. (1981). Guidelines for item selection in inventories yielding score profiles. *Educ. Psychol. Meas.* 41 1011–1016. 10.1177/001316448104100409

[B53] Milligan C. Littlejohn A. (2016). How health professionals regulate their learning in massive open online courses. *Internet High. Educ.* 31 113–121. 10.1016/j.iheduc.2016.07.005 27928198 PMC5125435

[B54] Moore R. L. Wang C. (2021). Influence of learner motivational dispositions on MOOC completion. *J. Comput. High. Educ.* 33 121–134. 10.1007/s12528-020-09258-8

[B55] Müller T. (2008). Persistence of women in online degree completion programs. *Int. Rev. Res. Open Distance Learn.* 9 1–18. 10.19173/irrodl.v9i2.455

[B56] Narayanasamy S. K. Elçi A. (2020). An effective prediction model for online course dropout rate. *Int. J. Distance Educ. Technol.* 18 94–110. 10.4018/IJDET.2020100106

[B57] Pérez-Álvarez R. A. Maldonado-Mahauad J. Sharma K. Sapunar-Opazo D. Pérez-Sanagustín M. (2020). characterizing learners’ engagement in MOOCs: an observational case study using the notemyprogress tool for supporting self-regulation. *IEEE Trans. Learn. Technol.* 13 676–688. 10.1109/TLT.2020.3003220

[B58] Pérez-Sanagustín R. Maldonado M. J. J. (2016). “How to design tools for supporting self-regulated learning in MOOCs? Lessons learned from a literature review from 2008 to 2016,” in *Proceedings of the 2016 XLII Latin American Computing Conference (CLEI)* (Valparaiso: IEEE), 1–12.

[B59] Pintrich P. R. De Groot E. V. (1990). Motivational and self-regulated learning components of classroom academic performance. *J. Educ. Psychol.* 82 33–40. 10.1037/0022-0663.82.1.33 24274775

[B60] Putwain D. W. Nicholson L. J. Pekrun R. Becker S. Symes W. (2019). Expectancy of success, attainment value, engagement, and achievement: a moderated mediation analysis. *Learn. Instr.* 60 117–125. 10.1016/j.learninstruc.2018.11.005

[B61] Romero-Rodríguez L. M. Ramírez-Montoya M. S. González J. R. V. (2020). Correlation analysis between expectancy-value and achievement goals in MOOCs on energy sustainability: profiles with higher engagement. *Interact. Technol. Smart Educ.* 17 417–434. 10.1108/ITSE-01-2020-0017

[B62] Schunk D. H. (2016). *Learning Theories an Educational Perspective*, 7th Edn. Sydney: Pearson Education.

[B63] Semenova T. (2022). The role of learners’ motivation in MOOC completion. *Open Learn. J. Open Distance e-Learn.* 37, 273–287. 10.1080/02680513.2020.1766434

[B64] Shea P. Bidjerano T. (2010). Learning presence: towards a theory of self-efficacy, self-regulation, and the development of a communities of inquiry in online and blended learning environments. *Comput. Educ.* 5 1721–1731. 10.1016/j.compedu.2010.07.017

[B65] Shin N. (2003). Transactional presence as a critical predictor of success in distance learning. *Distance Educ.* 24 69–86. 10.1080/01587910303048

[B66] Shukor N. A. Abdullah Z. (2019). Using learning analytics to improve MOOC instructional design. *Int. J. Emerg. Technol. Learn.* 14 6–17. 10.3991/ijet.v14i24.12185 27731844

[B67] Skinner E. A. Kindermann T. A. Connell J. P. Wellborn J. G. (2009). “Engagement and disaffection as organizational constructs in the dynamics of motivational development,” in Educational Psychology Handbook Series. Handbook of Motivation at School, eds Wenzel K. R. Wigfield A. (New York, NY: Routledge), 223–245.

[B68] Song J. Y. (2018). The role of gender in mathematics career choice, classroom engagement, and achievement–mathematics self-efficacy, task value, and task cost as mediators –. *J. Career Educ. Res.* 31 1–25. 10.32341/JCER.2018.06.31.2.1

[B69] Swan K. P. Richardson J. C. Ice P. Garrison D. R. Cleveland-Innes M. Arbaugh J. B. (2008). Validating a measurement tool of presence in online communities of inquiry. *Ementor* 2 88–94.

[B70] Turk M. Heddy B. C. Danielson R. W. (2022). Teaching and social presences supporting basic needs satisfaction in online learning environments: how can presences and basic needs happily meet online? *Comput. Educ.* 180:104432. 10.1016/j.compedu.2022.104432

[B71] Valle N. Antonenko P. Valle D. Dawson K. Huggins-Manley A. C. Baiser B. (2021). The influence of task-value scaffolding in a predictive learning analytics dashboard on learners’ statistics anxiety, motivation, and performance. *Comput. Educ.* 173:104432. 10.1016/j.compedu.2021.104288 PMC8153529 34075283

[B72] Wu B. Chen X. (2017). Continuance intention to use MOOCs: integrating the technology acceptance model (TAM) and task technology fit (TTF) model. *Comput. Hum. Behav.* 67 221–232. 10.1016/j.chb.2016.10.028

[B73] Wu F. Fan W. Arbona C. de la Rosa-Pohl D. (2020). Self-efficacy and subjective task values in relation to choice, effort, persistence, and continuation in engineering: an expectancy-value theory perspective. *Eur. J. Eng. Educ.* 45 151–163. 10.1080/03043797.2019.1659231

[B74] Yang B. Tang H. Hao L. Rose J. R. (2022). Untangling chaos in discussion forums: a temporal analysis of topic-relevant forum posts in MOOCs. *Comput. Educ.* 178 1–13. 10.1016/j.compedu.2021.104402

[B75] Yousef A. M. F. Sumner T. (2021). Reflections on the last decade of MOOC research. *Comput. Appl. Eng. Educ.* 29 648–665. 10.1002/cae.22334 32837579

[B76] Yusof A. Atan N. A. Harun J. Doulatabadi M. (2017). Understanding learners’ persistence and engagement in massive open online courses: a critical review for Universiti Teknologi Malaysia. *Man India* 97 147–157.

[B77] Zhang C. Chen H. Phang C. W. (2018). Role of instructors’ forum interactions with students in promoting MOOC continuance. *J. Glob. Inf. Manage.* 26 105–120. 10.4018/JGIM.2018070108

[B78] Zhao Y. Wang A. Sun Y. (2020). Technological environment, virtual experience, and MOOC continuance: a stimulus- organism-response perspective. *Comput. Educ.* 144:103721. 10.1016/j.compedu.2019.103721

[B79] Zhu M. (2022). Designing and delivering MOOCs to motivate participants for self-directed learning. *Open Learn. J. Open Distance e-Learn.* 1–20. 10.1080/02680513.2022.2026213

[B80] Zielinski M. West V. Merzdorf H. E. Douglas K. A. Bermel P. (2019). Motivation and perceived costs to achievement in advanced engineering MOOCs. *Int. J. Eng. Educ.* 35 1540–1550.

